# The impact of user fees on uptake of HIV services and adherence to HIV treatment: Findings from a large HIV program in Nigeria

**DOI:** 10.1371/journal.pone.0238720

**Published:** 2020-10-08

**Authors:** Aima A. Ahonkhai, Susan Regan, Ifeoma Idigbe, Olayemi Adeniyi, Muktar H. Aliyu, Prosper Okonkwo, Juliet Adeola, Elena Losina, Zaidat Musa, Oliver Ezechi, Kenneth A. Freedberg

**Affiliations:** 1 Division of Infectious Disease, Vanderbilt University Medical Center, Nashville, Tennessee, United States of America; 2 Vanderbilt Institute for Global Health, Vanderbilt University Medical Center, Nashville, Tennessee, United States of America; 3 Division of General Internal Medicine, Massachusetts General Hospital, Boston, Massachusetts, United States of America; 4 Nigerian Institute for Medical Research, Lagos, Nigeria; 5 Department of Health Policy, Vanderbilt University Medical Center, Nashville, Tennessee, United States of America; 6 APIN Public Health Initiatives (APIN), Abuja, Nigeria; 7 Medical Practice Evaluation Center, Massachusetts General Hospital, Boston, Massachusetts, United States of America; 8 Harvard Medical School, Boston, Massachusetts, United States of America; 9 Department of Orthopedic Surgery, Brigham and Women’s Hospital, Boston, Massachusetts, United States of America; 10 Department of Biostatistics, Boston University School of Public Health, Boston, Massachusetts, United States of America; 11 Divisions of Infectious Disease and General Internal Medicine, Massachusetts General Hospital, Boston, Massachusetts, United States of America; 12 Harvard University Center for AIDS Research (CFAR), Boston, Massachusetts, United States of America; 13 Department of Health Policy and Management, Harvard T.H. Chan School of Public Health, Boston, Massachusetts, United States of America; Ohio State University, UNITED STATES

## Abstract

**Background:**

Global HIV funding cutbacks have been accompanied by the adoption of user fees to address funding gaps in treatment programs. Our objective was to assess the impact of user fees on HIV care utilization and medication adherence in Nigeria.

**Methods:**

We conducted a retrospective analysis of patients enrolled in care before (October 2012-September 2013) and after (October 2014-September 2015) the introduction of user fees in a Nigerian clinic. We assessed pre- vs. post-user fee patient characteristics and enrollment trends, and determined risk of care interruption, loss to follow-up, and optimal medication adherence.

**Results:**

After fees were instituted, there was a 66% decline in patient enrollment and 75% decline in number of ART doses dispensed. There was no difference in the proportion of female clients (64% vs 63%, p = 0.46), average age (36 vs. 37 years, p = 0.15), or median baseline CD4 (220/ul vs. 222/uL, p = 0.24) in pre- and post-fee cohorts. There was an increase in clients employed and/or had tertiary education (24% vs. 32%, p<0.001). Compared to pre-fee patients, the post-fee period had a 48% decreased risk of care interruption (aRR = 0.52, 95%CI:0.39–0.69), 22% decreased LTFU risk (aRR = 0.64, 95%CI:0.96), and 27% decreased odds of optimal medication adherence (aOR = 0.7, 3 95%CI 0.59–0.89).

**Conclusions:**

Patients enrolled in care after introduction of user fees in Nigeria were more likely to be educated or employed, and effectively retained in care after starting ART. However, fees were accompanied by a drastic reduction in new patient enrollment, suggesting that many patients may have been marginalized from HIV care.

## Introduction

There are 37 million people living with HIV and AIDS worldwide [1], and 59% of this population now has access to life-saving antiretroviral therapy (ART). The success of the global AIDS response has relied heavily on funding from multilateral institutions and host-country governments [1, 2]. Since its inception in 2003, the US President’s Emergency Plan for AIDS Relief (PEPFAR) has contributed over $50 billion to this effort, the largest commitment by any nation to combat a single disease. This support has made free HIV care and treatment services available for patients in PEPFAR-supported countries, and in other resource-limited settings [2].

Originally developed to provide lifesaving ART in countries most burdened by HIV, in 2008 PEPFAR entered a new era, coined PEPFAR 2.0, shifting its approach from emergency response to sustainability and country ownership [3]. Following this transition, PEPFAR worked with recipient countries to increase their commitment to their own HIV programs. Nigeria, the most populated African country, is also home to the second largest population of people living with HIV worldwide. In the wake of PEPFAR 2.0, between 2011 and 2015, PEPFAR’s funding to Nigeria decreased by about $83 million in yearly program support [4]. While the Nigerian government committed to increase its funding contribution to its own national HIV/AIDS program from 7% of the total budget in 2008 to 50% by 2015, this commitment has been thwarted by fiscal challenges and inconsistent political will at the state and local government levels [5, 6]. In 2016, Nigeria had the poorest performing economy in Sub-Saharan Africa; and as future funding remains uncertain for Nigeria the gap between available resources and anticipated need may widen [2, 7].

Early on in the global AIDS response, many patients were required to pay out-of-pocket (OOP) expenses for ART, clinical services, and transportation [8–10]. Meta-analyses of low and middle-income countries reported that clinics with user fees had 30% fewer patients achieving virologic suppression, and a 4-fold increased risk of attrition and death [9–11]. Even in the setting of highly subsidized HIV care, interruptions in care are common in resource-limited settings [12]. Patients cite a wide range of reasons for missing clinic visits, including indirect healthcare costs, such as transportation fees and lost wages [13, 14]. While some interruptions from care are prolonged, or even indefinite, reports from Nigeria suggest that brief interruptions in care occur in at least 1 of 3 patients [12, 15]. These interruptions are associated with halving of the expected gains in CD4 numbers usually seen with ART [16].

In the Nigerian setting, the gap between multinational donor support and government contribution to HIV care has been met, in many clinics, by charging fees to patients. Little is known about how patients will respond to these user fees after a decade of free care. Given the data from the early ART era on the deleterious impact of user fees, and the high rates of care interruption in the setting of free care, we hypothesized that the introduction of user fees in Nigeria would be associated with less engagement in care and poorer medication adherence. The objective of this analysis, therefore, is to assess the impact of introducing patient user fees for HIV care on individual patient care utilization and medication adherence in a large HIV clinic in Lagos, Nigeria.

## Methods

### Setting

This study was conducted at the Nigerian Institute for Medical Research (NIMR). NIMR is a President’s Emergency Plan for AIDS Relief (PEPFAR)-supported clinic located in Lagos, Nigeria. With a population of more than 13 million, Lagos is Nigeria’s biggest city and one of PEPFAR’s priority states for effective ART scale-up [17]. NIMR began providing HIV care in 2002 and currently has 7,351 patients enrolled in care. Patient user fees were instituted in October 2014; children and pregnant women were exempt from payment. The fee schedule is expressed in 2015 USD and includes HIV testing (3 USD), new patient consultation (25 USD), new patient labs (45 USD), and follow up consultations (10 USD), and routine labs (14 USD). Routine labs included white blood cell count, platelet count, ALT, creatinine, Hgb. In total, yearly user fees at NIMR are 166 USD, but 82% of Nigerians live on less than 2 USD per day [Table 1] [18, 19].

**Table 1:** Fees for HIV care in NIMR^α^ clinic.

Costs in USD^β^	Unit Cost ($)	Yearly Cost ($)
**HIV Test**	3	3
**New Patient Consultation**	25	25
New Patient Labs*	45	45
**Consultation Fee**	10	20
**ART Pick-Up**	5	72
**Routine Labs** (WBC^δ^, Platelets, ALT^ε^, Cr^θ^, Hgb, CD4, HIV RNA)	14	28
**Total (USD)**		**$190**

^α^ Nigerian Institute for Medical Research.

^β^ United States Dollar

^δ^ White Blood Cell.

^ε^ Alanine Aminotransferase

^θ^ Creatinine.

### Study design

We conducted a retrospective cohort study comparing two patient groups. The pre-user fee cohort consisted of patients who enrolled in care at NIMR and initiated ART between October 1, 2012 and September 30, 2013, and were followed through September 30, 2014. The post-user fee cohort consisted of patients who enrolled in care at NIMR and initiated ART between October 2014 and September 30, 2015, (after the institution of user fees), and were followed through September 30, 2016. The exposure of interest was the receipt of HIV care in the user fee era at NIMR. We assessed three patient outcomes 12 months after clinic enrollment: 1) care interruption, defined as having a period 90 days or more without a clinic, pharmacy or laboratory visit, but with return to care within the 12-month follow-up period; 2) loss to follow-up (LTFU), defined as having a period of 90 days or more without a clinic, pharmacy or laboratory visit, but without return to care within the 12-month follow-up period; and 3) an adapted measure of the medication possession ratio (MPR). The traditional MPR is defined as the number of daily doses of ART dispensed divided by the total number of drug pick-ups over the follow-up period. However, medication surplus in some periods can negate missed doses in other periods.

**Fig 1:**
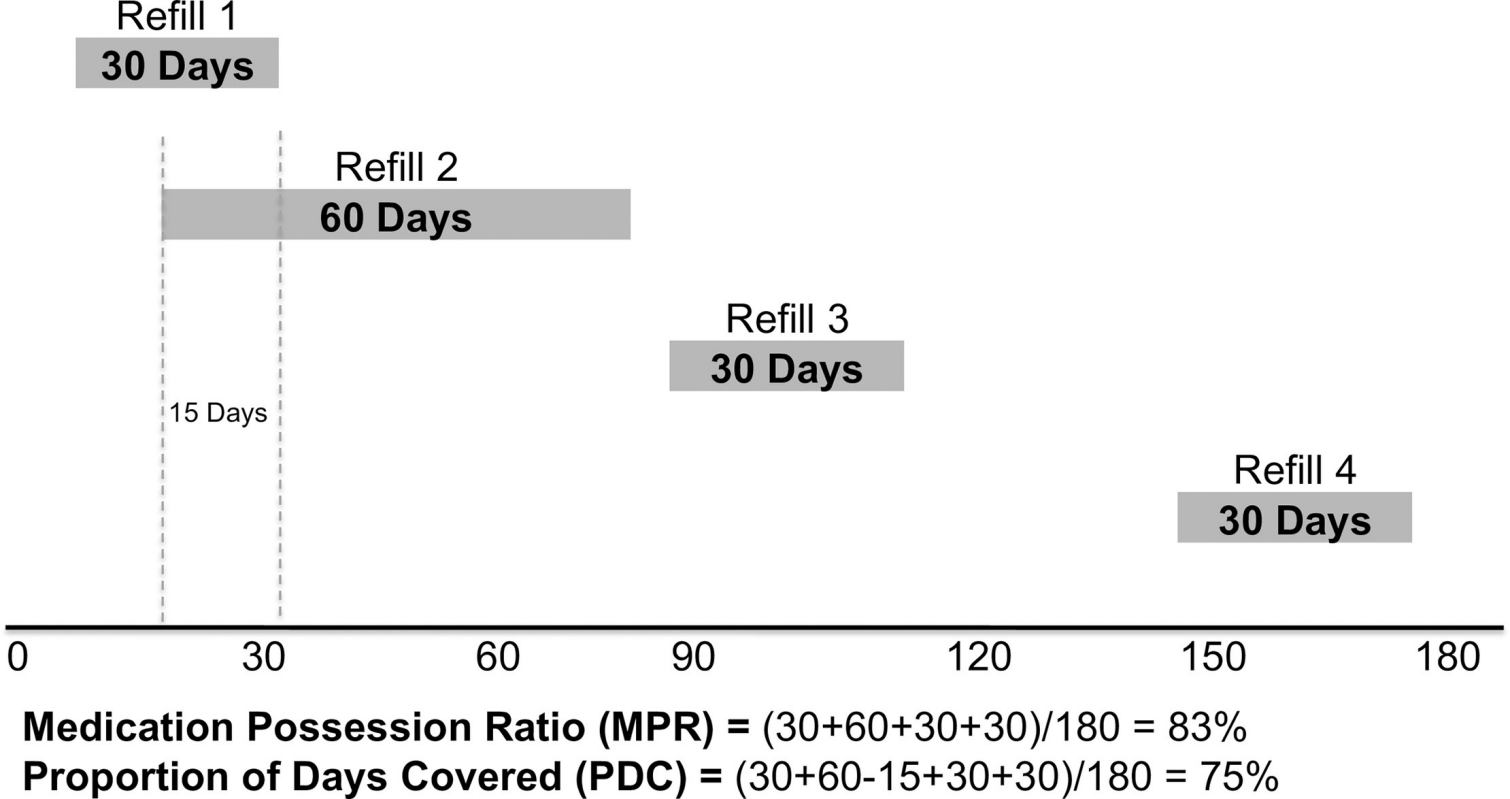


The proportion of days covered (PDC) is a pharmacy-based method of calculating adherence that, unlike the medication possession ration (MPR), ensures that missed doses during one pharmacy refill interval are not negated by medication surplus (defined as overlap between two dispenses) in another pharmacy refill interval. PDC is calculated by first multiplying the number of refills obtained by the number of days prescribed for each refill, then subtracting the number of days in which there was an overlap of ART filled between refill periods, and finally dividing this total by the number of days in the follow-up period [20]. A schematic of this measure is seen in Fig 1. The PDC measure can never be greater than 100%, and never more than the MPR. The PDC was categorized as optimal (>94%), suboptimal (80–94%), and poor (<80%), based on existing literature associating these categories of MPR with the risk of virologic failure [21].

### Data collection and analysis

All data, including baseline demographic information, clinical, laboratory, and drug pick-up visits were abstracted from standardized data entry forms used for data management across the country. These data entry forms were usually validated and entered daily into NIMR’s central electronic clinical database, which is managed by a team of data mangers. After abstraction, the data were further cleaned before data analysis.

Yearly enrollment and HIV testing trends at NIMR were summarized in the two years prior to and one year after the introduction of user fees at NIMR in October 2014. Baseline demographics including age, sex, marital status, and a variable termed “high earner” (having tertiary education and/or being employed), and clinic characteristics (baseline CD4 count) of the pre- and post-fee cohorts were also summarized. Chi-square tests (for categorical variables) and Kruskal-Wallis tests (for ordinal variables) were used to assess whether baseline characteristics differed by patient cohort.

Multinomial, multivariable logistic regression was used to assess the impact of user fees on interruptions from care and LTFU. Potential confounders (of the relationship between user fees and care interruption or LTFU) and other independent risk factors for care interruption and LTFU, including sex, marital status, age, education, employment, high earner status, and baseline CD4 count were adjusted for. To determine the impact of user fees on medication adherence, chi square tests was used to assess for differences in adapted MPR categories in the pre- and post-user fee cohorts. Furthermore, multivariable logistic regression was used to measure the association between user fee period and the odds of having poor or suboptimal medication adherence (defined by having PDC <94% over 12 months of follow-up). Finally for both models, likelihood ratio tests was used to determine whether inclusion of education, employment, or high earner status provided the best fit in multivariate analysis.

### IRB approval

We obtained IRB approval from Partners HealthCare (Protocol no. 2015P001480 Boston, MA, USA) the Nigerian Institute for Medical Research (Protocol no. IRB/15/316) in Lagos, Nigeria, and Vanderbilt University Medical Center (Protocol no. 161779 Nashville, TN, USA). All data used for the purpose of this research were fully anonymized prior to access by the study team, and a waiver for informed consent was obtained from the Partners HealthCare, Nigerian Institute for Medical Research, and Vanderbilt University IRB committees.

## Results

### Trends in enrollment and HIV testing before and after user fees

**Fig 2:**
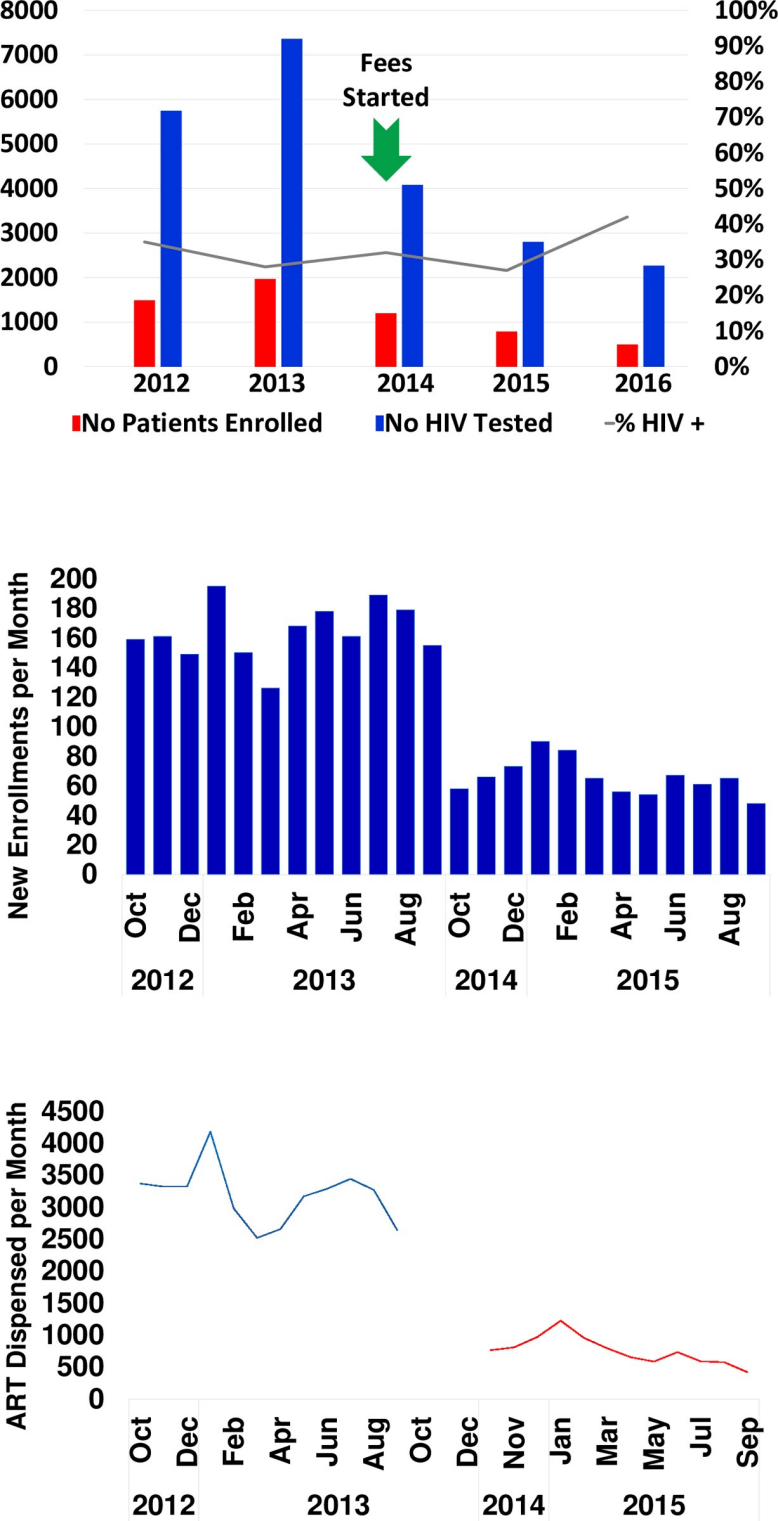
Declines in patient enrollment, HIV testing, and ART dispensing after user fees instituted in a Nigerian clinic. This shows the number of patients enrolled in the HIV clinic by year, as well as the number of HIV tests performed, and proportion testing positive. (A) Yearly trends in HIV testing and patient enrollment (B) Monthly trends in new patient enrollment (C) Monthly trends in doses of ART dispensed. No = Number.

Yearly enrollment of new patients was greater in the years before user fees were instituted (2012: 1,490 patients, 2013: 1,970 patients, 2014: 1,200 patients), than in the years after user fees were instituted (2015: 787 patients, 2016: 499 patients.) Between 2014 and 2015, there was a 66% decline in new patient enrollment each year [Fig 2, Panel A]. The declining trend in new patient enrollment was mirrored by the number of ART doses dispensed each month by the pharmacy [(Fig 2, Panels B and C]). When assessed on a monthly time scale, average monthly patient enrollment declined from 164 patients/month to 66 patients/month in the pre- versus post-user fee eras, and the average number of ART doses dispensed declined from 3,182 to 757 doses. A similar pattern was seen in HIV testing, with the number of patients tested greater before user fees were charged (2012: 5,745 tested, 2013: 7,360 tested, 2014: 4,082 tested) than after (2015: 2,801 tested, 2016: 2,265 tested). The proportion of individuals who tested positive for HIV did not follow the same pattern and ranged from 27% to 42% during the same period [Fig 2].

### Description of pre- and post-user fee cohorts

A total of 2,757 patients were included in this analysis. Majority of the patients 1,970 (71%) were in the pre-user fee cohort who enrolled in care between September 2012 and September 2013, and 787 patients (29%) in the post-user fee cohort who enrolled in care between October 2014 and October 2015. There was no difference in the proportion of female patients (64% vs. 63%, p = 0.46) or the mean age (36.4 vs. 37.0 years, p = 0.15) in the two cohorts (Table 2). There was no statistically significant difference in the proportion of patients who were employed (82% vs 85%, p = 0.10), but a significant increase in those who had secondary or tertiary education (74% vs. 80%, p<0.001) after user fees were instituted, and also a greater absolute difference was seen in the “high earner” category of patients (those who were employed and/or had tertiary education) pre- vs. post-user fee (24% vs. 32%, p<0.001). There was no significant difference in the proportion of patients with a baseline CD4 count <200 in the pre- vs. post- user fee cohorts (46% vs. 47%, p = 0.12) nor in the median baseline CD4 count at clinic enrollment (220cells/uL vs. 222cells/uL) cohorts (p = 0.24).

**Table 2:** Baseline demographics of pre- and post-user fee cohorts in a large Nigerian clinic.

		Pre-User Fee n = 1970	Post-User Fee n = 787	P value
**Female**		1,261 (64%)	492 (63%)	0.446
**Age (years)**	Mean	36	37	0.15
**Employed**		1,611 (82%)	664 (85%)	**0.510**
**Education**	None	116 (6%)	28 (4%)	**0.001**
	Primary	396 (20%)	126 (16%)	
	Secondary	819 (42%)	328 (42%)	
	Tertiary	637 (32%)	301 (38%)	
**High Earner**	Employed and/or Tertiary Education	474 (24%)	251 (32%)	**<0.001**
**CD4 Count**	Median (IQR)^α^	220 (88–413)	222 (85–386)	0.239

^α^ Interquartile Range.

### Care interruption and Loss-To-Follow-Up (LTFU) before and after user fees

More patients in the post-user fee cohort were retained in clinic without any care interruption (57% vs. 49%), defined as 90 days or more without clinic, pharmacy, or laboratory visit; with fewer patients having one or more interruptions in care (12% vs. 18%) or were lost to follow-up (31% vs. 33%) compared to the pre-user fee cohort (p<0.001). These findings persisted in a multinomial model adjusting for sex, marital status, age, employment, education, and baseline CD4 count. Of note, inclusion of both education level and employment status (rather than high earner status, or any if these variables alone) provided the best model fit). Compared to those unemployed, employed patients [aRR = 0.78, 95%CI 0.59–1.03] and students [aRR = 0.28, 95%CI 0.16–0.49] had a reduced risk of LTFU, but did not reach statistical significance for care interruption. Similarly, compared to patients with no education, those with primary [aRR = 0.83, 95%CI 0.54–0.26], secondary [aRR = 0.49, 95%CI 0.33–0.74], or tertiary [aRR = 0.35, 95%CI 0.24–0.53] education had a reduced risk of LTFU but not care interruption. Adjusting for these factors, patients in the post-user fee cohort had a reduced risk of care interruption [aRR = 0.52, 95%CI:0.39–0.69] and LTFU [aRR = 0.78, 95%CI:0.64–0.96] compared to those in the pre-user fee cohort [Table 3].

**Table 3:** Risk of care interruption or loss to follow-up after institution of user fees in a Nigerian clinic.

		Interruptions in Care ^α^		Lost to Follow Up	
		aRR^β^	95% CI*	aRR^β^	95% CI
**Cohort**	Pre-User Fee Cohort (reference)	1.00		1.00	
	Post User Fee Cohort	0.52	0.39–0.69	0.78	0.64–0.96
**Sex**	Male (ref)	1.00		1.00	
	Female	097	0.75–1.26	0.73	0.60–0.88
**Marital Status**	Unmarried (reference)	1.00		1.00	
	Married	0.87	0.68–1.12	0.81	0.67–0.98
**Age Category**	>30 years	1.00		1.00	
	30–34 years	0.84	0.59–1.20	0.80	0.60–1.06
	35–44 years	0.91	0.65–1.28	0.88	0.67–1.14
	≥45 years	0.87	0.59–1.28	0.73	0.54–0.99
**Baseline CD4 <200cells/uL**	0–200 (reference)	1.00		1.00	
	201–350	1.06	0.73–1.52	0.50	0.40–0.64
	351–500	6.18	4.40–8.68	1.10	0.82–1.48
	>500	17.43	12.31–24.77	3.77	2.81–5.06
	Unknown	9.21	4.65–18.22	9.51	5.74–15.78
**Employment**	Unemployed (reference)	1.00		1.00	
	Employed	1.44	0.97–2.15	0.78	0.59–1.03
	Student	1.00	0.54–1.85	0.28	0.16–0.49
**Education**	None (reference)	1.00		1.00	
	Primary	1.64	0.83–3.26	0.83	0.54–1.26
	Secondary	1.08	0.58–2.08	0.49	0.33–0.74
	Tertiary	1.28	0.66–2.47	0.35	0.24–0.53

^α^ Multimodal model with no interruptions in care as the reference group.

^β^ Adjusted Risk Ratio.

* 95% Confident Interval.

### ART initiation before and after user fees

In the post-user fee cohort, only 19% of patients (n = 152) did not start ART in their first year after enrollment, compared to 32% (n = 638) of patients in the pre-user fee cohort (p<0.001). However, among those patients who did start ART, the median time to the first ART pick-up was shorter in the post-user fee cohort (35 days: IQR 20–50) than in the pre-user fee cohort (45 days: IQR 28–63, p<0.001). The proportion of patients with optimal adherence, defined as PDC >94%, was greater in the pre-user fee cohort (61% vs. 52%). The proportion of patients with suboptimal (PDC 80–94%) and poor (PDC <80%) adherence was greater in the post-user fee cohort (23% vs 18%; 27% vs. 22% respectively; p overall <0.001) [see S1 Fig, which shows the percent of patients with optimal, sub-optimal, or poor adherence in each cohort]. In multivariable analysis after adjusting for sex, marital status, age, high earner status, and baseline CD4 count, patients in the post-user fee cohort had reduced odds of having optimal adherence compared to those in the pre-user fee cohort (aOR = 0.7, 3 95%CI 0.59–0.89) [Table 4]. In contrast to the model assessing care interruption and LTFU before and after user fees [Table 3], inclusion of the high earner variable alone (rather than employment and/or education, provided better fit for this model and so was retained in the final iteration. Notably, those who were high earners had a 25% increased odds of suboptimal adherence.

**Table 4:** Odds of having optimal adherence* after institution of user fees in a Nigerian clinic.

		aOR^α^	95% Confidence Interval
**Cohort**	Pre-User Fee Cohort (reference)	1.00	0.59–0.89
	Post User Fee Cohort	0.73	
**Sex**	Male (reference)	1.00	0.97–1.43
	Female	1.18	
**Marital Status**	Unmarried (reference)	1.00	0.68–1.00
	Married	0.83	
**Age Category**	≤30 years (reference)	1.00	
	30–34 years	1.18	0.80–1.71
	35–44 years	1.25	0.85–1.85
	≥45 years	1.10	0.73–1.65
High Earner*	No (reference)	1.00	1.02–1.55
	Yes	1.26	
**Baseline CD4 Stratum**	≤200 cells/uL (reference)	1.00	
	201–350 cells/uL	1.21	0.97–1.50
	351–500 cells/uL	1.07	0.80–1.43
	≥500 cells/uL	0.78	0.54–1.12
	Unknown	0.32	0.15–0.70

^α^ Adjusted Odds Ratio.

* High Earner Status: tertiary education and/or employed.

## Discussion

Our analysis of a large PEPFAR-supported HIV program in Nigeria highlights concerning important trends in the utilization of HIV treatment services in the wake of the introduction of patient user fees. This is not a novel concern, as the role of user fees for healthcare in resource-limited settings have been widely debated [22, 23]. While some have argued that user fees decrease excessive healthcare use, and create resources to help improve the quality of clinical services, others argue that user fees are inequitable, and prevent the poorest of patients from accessing necessary healthcare services [23, 24]. In our study setting, user fees were adopted in response to funding cutbacks from important donors, such as PEPFAR. Indeed, the transition to PEPFAR 2.0. has called for increased country ownership and investment in the local HIV response. Without the backdrop of financial solvency and political commitment to address HIV care and treatment needs at the country level, however, administrators of treatment programs appear to have been left to meet the gap between multinational contributions, local government funding, and programmatic costs.

In 2015, the year after user fees were instituted, there was a sharp and persistent decline in HIV testing, and new patients enrollment, at NIMR. The dramatic decline in new patient enrollment was additionally accompanied by a 4-fold decline in the number of cumulative monthly doses of ART dispensed by the clinic pharmacy. While we cannot conclude that user fees alone were responsible for this decline, anecdotal evidence of the same trend has been observed at other sites throughout Nigeria, and the magnitude of the decline is consistent with data from other studies [24]. Another study from our investigating the impact of PEPFAR policy changes and accompanying funding cutbacks in a large treatment network in Nigeria underscores that charging of user fees were a symptom of a larger problem. Diminishing resources for staff salaries and training, IT support, HIV outreach services, were also observed over the same period. Nonetheless, data from a Cochrane review evaluating 16 studies on the impact of user fees on health services in low- and middle-income countries showed that use of both preventive and curative healthcare services decreased, typically in one large step-down, when user fees were introduced or increased as we also observed [22, 23]. The dramatic decline in new patient enrollment was additionally accompanied by a 4-fold decline in the number of cumulative monthly doses of ART dispensed by the clinic pharmacy among newly enrolled patients during the same period. This trend underscores the potential community-level impact of having fewer patients in care and fewer on ART, which in turn can create important barriers to epidemic control.

Our findings also support the notion that user fees may create a crucial barrier to access for patients with the fewest resources [24, 25]. When comparing the cohort of patients who received care before and after user fees were instituted, there were no significant differences in age, sex, or baseline CD4+ cell count between the two patient groups. However, patients who enrolled in care after user fees were established were more likely to be well-resourced, having either been employed or completed some college-level education. While the policy of the clinic at NIMR was to make exemptions for the poorest patients, our findings suggest that this policy was not sufficient to overcome the financial obstacles for many. Besides, other data suggest that exemptions often do not work to eliminate these obstacles for poor patients [24].

Despite the dramatic decline in new patient enrollment after user fees were instituted, our analysis highlights improvement in retention in care in the post user-fee era. Indeed, patients who had to pay for care had a 45% reduction in the risk of interrupting care, and a 23% reduction in the risk of being lost to follow-up, compared to patients in the pre-user fee cohort. Some data on user fees suggest that utilization may increase if fees are accompanied by an increase in the quality of healthcare services provided [23, 26, 27]. However, without concerted efforts to improve the quality of services, utilization drops disproportionately for the poorest patients due to the compounding effect of direct (i.e. user fees) and indirect costs, the most costly of which is often transportation [24, 26, 27]. As a result, poor patients may delay seeking care until they are sicker [26]. Our findings did not directly support this assertion, as there was no change in the median CD4+ cell count at enrollment in our pre- and post-user fee cohorts, although CD4+ cell count at presentation was fairly low in both groups. In addition, the fact that retention in care improved for the entire post user-fee cohort, 68% of whom were unemployed or had high school or less education, suggests that additional factors might be at play. Retention in care has been a major barrier to effective HIV care delivery globally, even in the setting of free care [16, 28–30]. Our findings of improved retention and care utilization in the post-fee era may highlight an association between personal financial investment in care and clinic attendance.

We found that patients who paid fees were not only less likely to have interruptions in care or become lost to follow-up, but also more likely to have started ART at all. Despite these positive health-seeking behaviors, patients who paid for ART were less likely to have optimal medication adherence, as assessed by medication pick-up from the pharmacy in the year after starting ART. The reasons for this are not entirely clear. Monthly ART costs are the equivalent of 2.75 USD, but about 80% of Nigerians subsist on less than 2 USD per day [19]. It is possible, therefore, that the required monthly ART pick-up in the first year on treatment may have imposed additional, prohibitive indirect healthcare costs from transportation and lost wages [19]. Such costs could disproportionately affect employed patients who might have lost wages with each visit to the pharmacy to pick up ART, or who might not have been given permission to leave work for pharmacy visits. Alternatively, employed patients who might have been inconvenienced by ART pick-up at the clinic pharmacy could have chosen to pay for unsubsidized ART at a local pharmacy without having to wait in long lines or miss time from work. Data on healthcare fees show that in addition to user fees, out-of-pocket medication costs also pose a barrier to medication adherence [27, 31, 32]. One study from Nigeria conducted when HIV care was free to patients found that out-of-pocket expenses for HIV care accounted for 40% of healthcare expenses–a threshold that meets the WHO’s definition of financial catastrophe [33, 34]. Other data from Cote d’Ivoire highlights that the risk of financial catastrophe decreases with increasing time in ART, further underscoring the need to find sustainable and equitable strategies to initiate those living with HIV on ART [35].

Some programs have instituted protections to limit the burden of out-of-pocket expenses imposed by charging user fees [36]. NIMR (our study site), for example, instituted fee exemptions for children, pregnant women, elderly and indigent patients. Additional options include less intensive monitoring for stable patients. There are also many opportunities to greatly decrease the cost of HIV care such as Nursing Centered care instead of Physician Centered care which has been shown to be effective in studies in South Africa, with less intensive monitoring, and potentially less expensive procurement of ART [37–39]. However, the reliance on user fees for HIV care is not unique in Nigeria. Similar user fee-based strategies have been implemented in other countries the region, and particularly in Western Africa, to cover the cost of lab monitoring tests, hospital registration clinical consultations, as well as to compensate for low provider wages [40]. Mean yearly fees vary widely depending on context, average around $40 USD yearly, and range from as low as $4 USD to as much as $166 USD [36, 40]. There is a global challenge to dramatically decrease the cost of ART care and data such as these can help drive the urgency of this challenge.

Our study has some important limitations. The retrospective and pre-post study design prevents us from being able to establish a causal relationship between the introduction of user fees and our study outcomes. Our study was also limited to one clinical site and is not necessarily generalizable to other settings. In addition, while we did not formally quantify the impact of temporal events that could also have contributed to our findings, we have some reassurance that important economic and programmatic changes would not likely negate our findings. On the economic side, inflation rates in Nigeria reached a 5-year low during the period of transition to user fees, suggesting a more favorable economic climate to respond to healthcare expenses. On the programmatic side, a coordinated effort encouraged by PEPFAR to down-refer stable patients at tertiary facilities (such as NIMR) to community-based clinics occurred most aggressively in the pre-user fee period, and therefore does not explain the declines in enrollment that we observed [41].

In conclusion, introducing user fees for HIV care in a Nigerian clinic may largely impact enrollment of new patients into clinic, as suggested by the substantial (nearly half) decrease at our study site. We observed improved retention in care among the patients who did enroll in care after fees were instituted, perhaps driven by the investment in care necessitated by the payment of user fees. However, this finding was mitigated by poorer adherence to ART among the same patients. These trends in care utilization and medication adherence are alarming, and stand in stark contrast to local and global efforts to reach UNAIDS 90-90-90 targets [1, 42]. In the case of NIMR and other clinical sites in Nigeria, user fees were introduced to meet critical funding gaps; but other data suggest that such fees are usually ineffective for healthcare financing, meeting at most 5–10% of recurrent budgetary requirements [24]. Our findings highlight the urgent need for creative, sustainable financing options that eliminate user fees and instead leverage mechanisms for pre-payment and risk-pooling, to support HIV care in Nigeria and other resource-limited settings [35].

## Supporting information

S1 Fig Optimal medication adherence decreases in the post-user fee era. This shows the percent of patients with >94% (optimal), 80–95% (sub-optimal), or <80% (poor adherence in the pre- vs. post-user fee cohort).
(TIF)
