# Supplementary figures and images for: The impact of user fees on uptake of HIV services and adherence to HIV treatment: Findings from a large HIV program in Nigeria
Source: PLoS One. 2020 Oct 8;15(10):e0238720. doi: 10.1371/journal.pone.0238720 (PMC7544141; doi:10.1371/journal.pone.0238720)

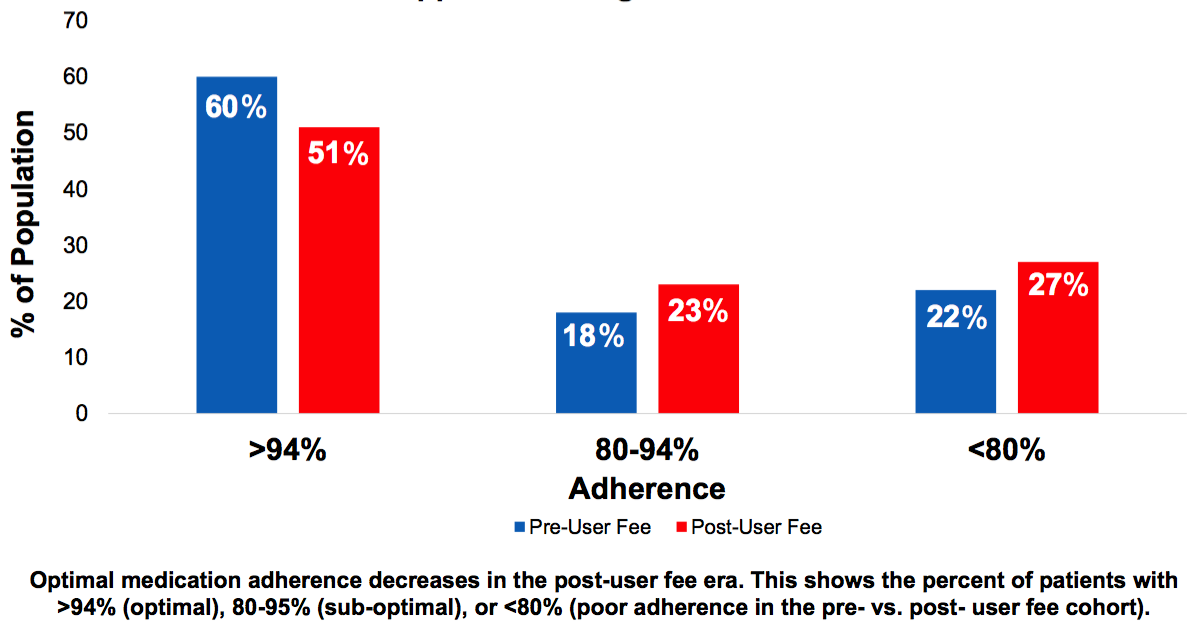

Supplement: S1 Fig — This shows the percent of patients with >94% (optimal), 80–95% (sub-optimal), or <80% (poor adherence in the pre- vs. post-user fee cohort). (TIF) [file pone.0238720.s001.tif]
